# Determinants of Telehealth Service Use among Mental Health Patients: A Case of Rural Louisiana

**DOI:** 10.3390/ijerph19116930

**Published:** 2022-06-06

**Authors:** Monteic A. Sizer, Dependra Bhatta, Binod Acharya, Krishna P. Paudel

**Affiliations:** 1 Northeast Delta Human Services Authority, 2513 Ferrand Street, Monroe, LA 71201, USA; monteic.sizer@la.gov; 2 Urban Health Collaborative, Drexel University, 3600 Market Street, Philadelphia, PA 19104, USA; ba525@drexel.edu; 3 Economic Research Service, United States Department of Agriculture, 805 Pennsylvania Avenue, Kansas City, MO 64105, USA; krishna.paudel@usda.gov

**Keywords:** mental health, visit intensity, telehealth, rural, COVID-19

## Abstract

The COVID-19 pandemic decreased the in-person outpatient visits and accelerated the use of telehealth services among mental health patients. Our study investigated the sociodemographic and clinical correlates of the intensity of telehealth use among mental health patients residing in rural Louisiana, United States. The study sample included 7069 telehealth visits by 1115 unique patients encountered from 1 April 2020 to 31 March 2021 at six mental health outpatient clinics managed by the Northeast Delta Human Services Authority (NEDHSA). We performed a negative binomial regression analysis with the intensity of service use as the outcome variable. Being younger, female, and more educated were associated with a higher number of telehealth visits. The prevalence of other chronic conditions increased telehealth visits by 10%. The telehealth service intensity varied across the nature of mental health diagnoses, with patients diagnosed with the schizophrenia spectrum and other psychotic disorders utilizing 15% fewer telehealth visits than patients diagnosed with depressive disorders. The promotion of telehealth services among mental health patients in the rural setting might require the elimination of the digital divide with a particular focus on the elderly, less educated, and those with serious mental health illnesses such as schizophrenia and psychotic disorders.

## 1. Introduction

Mental health disorder is one of the leading causes of disability and morbidity globally and in the United States [1,2]. Although mental health disorders are largely preventable and treatable [3,4,5], nearly one in five U.S. adults live with mental health challenges [6,7]. Since mental illnesses may result in low quality of life, high mortality rate, limited work productivity, and deprivation of social networks within the community [8], improving access to and quality of mental health services remains a key priority area of national and local governments. Notwithstanding the goal of public health institutions to promote health equity, disparities related to the access and utilization of mental health care services persist. In particular, mental healthcare utilization among rural residents is lower than in urban populations [9]. Prevailing stigma, lower financial resources, limited access to transportation and internet services, and inadequate mental health treatment facilities and practitioners are some of the reasons for the lower utilization of mental health services in the rural region [10,11,12].

The grief and physical isolation during the COVID-19 pandemic exacerbated the psychological distress to an unprecedented level, raising alarms about the mental health conditions across the country. At the same time, the government undertook multiple measures in containing the spread of the virus including the declaration of a nationwide emergency and the implementation of stay-in-home policies that actively discouraged in-person medical visits for non-life-threatening conditions [13] and mass vaccinations [14]. With the enactment of the CARES act in March 2020, healthcare providers responded to this disruption in healthcare service by expanding telehealth coverage, aimed at substituting the reduction in in-person visits by telephone and video-based engagements with the patients. Telehealth services also became available for mental health patients, which was a group that was shown to have promising results with telemedicine in the pre-pandemic period [15]. Although the effectiveness of telehealth services during the pandemic remains an area of active research, the emerging evidence implies that the shifts to telemedicine service were not sufficient to fill the gap in in-person visits [16]. Given that the residents in rural areas face additional barriers in utilizing telemedicine services arising from the existing disparities in broadband connectivity and technology literacy [17], it is plausible that rural patients may be disproportionately affected by the switch from in-person to telemedicine. Furthermore, past works imply that telemedicine may have a varying degree of success across diagnoses [18]. Even among mental health patients, the effectiveness of telehealth visits to patients with serious mental health issues such as schizophrenia and bipolar disorder remains questionable [18]. This highlights the need for studies examining the different facets of telehealth services and their utilization among mental health patients, particularly in rural settings.

A central focus of the public health and clinical research on mental health has been on improving healthcare utilization, which might be affected by various factors such as socioeconomic context, health attitudes and beliefs, and physical and mental status of health [19]. Andersen’s behavioral model of healthcare use conceptualized the determinants of healthcare utilization as *predisposing*, *enabling*, and *need* factors [20,21]. The *predisposing* factors are centered on sociodemographic characteristics (for example, gender, age, race, and education) and attitude toward the treatment or illness. The *enabling* factors include financial and organizational resources including health care accessibility components such as income, community resources, social support, and service availability. The *need* factors capture the illness-related factors such as the co-occurrence of multiple chronic conditions and type and nature of the illness. *Predisposing*, *enabling*, and *need factors*, together, are important in explaining and predicting healthcare service use. Knowledge about the relative contribution of these factors might help inform the state of disparity in healthcare service use and might provide guidance on the best course of public health action [20].

In this paper, we explore the dynamics of telemedicine service utilization by mental health patients encountered during the COVID-19 pandemic in a health system serving rural communities in Louisiana, which is a state with relatively poor overall health and quality of life [22]. In particular, we examine the mental healthcare use intensity, proxied by the number of visits for mental health reasons, in a healthcare system serving mostly rural parishes of Louisiana. We use the intensity of treatment because it is a more dynamic indicator of disease burden [23] and has added advantage over using a dichotomous response of whether an individual utilized the service or not [8,24,25,26]. This study will describe the pattern of and variations in telehealth service use in rural Louisiana and explore the factors associated with the intensity of treatment. Understanding the mental health care use intensity and the variation in health care utilization by different subgroups of mental health patients is important to frame clinical guidelines and policy decisions and allocate the mental healthcare resources optimally.

## 2. Materials and Methods

### 2.1. Study Region

Our research is focused on the twelve, mostly rural parishes (equivalents of counties in other U.S. states) of northeast Louisiana: Union, Lincoln, Jackson, Ouachita, Caldwell, Morehouse, Richland, Franklin, Tensas, Madison, East Carroll, and West Carroll. The region has a high prevalence of food insecurity, poverty, and mental health challenges [27]. The mean annual median household income in the study region was $37,371, which was lower than the rest of the parishes in Louisiana (Table 1). Only 62% of the households in the region had an internet subscription. Compared to the rest of Louisiana, the study region has a higher proportion of African American and elderly population.

### 2.2. Study Setting and Population

The Northeast Delta Human Services Authority (NEDHSA) is one of the local government entities of the Louisiana Department of Health and provides healthcare services to individuals with mental health challenges, developmental disabilities, and addictive disorders. NEDHSA serves the residents of northeast Louisiana through its six outpatient behavioral health clinics, which are located in Caldwell, Morehouse, Ouachita, Lincoln, Madison, and Franklin parishes. Trained health professionals working for NEDHSA provide services in the outpatient clinics. This study uses patients aged 18 years or older who received psychiatric care in NEDHSA outpatient clinics between 1 April 2020 and 31 March 2021. Out of the 7105 total services that NEDHSA provided during the study period, only 36 visits were physical visits, and thus, the associated patients were excluded from the analysis. A total of 7069 telehealth visits by 1115 unique patients were included in this study. Patient demographics, primary diagnosis, service date and type, number of visits, and discharge were obtained from NEDHSA’s electronic health record system. The Diagnostic and Statistical Manual of Mental Disorder, Fifth Edition (DSM-5-TR) was used to classify mental illness diagnosis and reason for visits. Informed consent was obtained from patients, and all study procedures were reviewed and approved by the Institutional Review Board of the Louisiana Department of Health.

### 2.3. Empirical Model and Statistical Analysis

The intensity of visit, defined as the number of total visits by a patient during the study window, is the dependent variable. Because the outcome is a count-variable, the conventional way of modeling it is by the Poisson point process [28] such that the number of visits follows a Poisson distribution with density [29]:

(1) $$P\left(Y=y\right)=\frac{\exp\left(-\lambda \right)\lambda^{y}}{y!},$$

y=0, 1, …,m;

where Y is the random variable that represents the number of clinic visits and λ is the rate parameter.

In Poisson distribution, the first moment (mean) should be equal to the second moment (variance):

(2) E(Y)=var(Y)=λ 

However, the equidispersion property of Poisson distribution often becomes violated in a real scenario [28]. In the case of overdispersion, Negative Binomial distribution performs better [28,30,31]. The first and second moments of Negative Binomial distribution are given as mean, $E\left(Y_{NB}\right)=\lambda$ and variance,

(3) $$var\left(Y_{NB}\right)=\lambda +\phi \lambda^{2},$$

where the parameter ϕ is related to overdispersion. If it equals zero, then the variance of the negative binomial distribution is equal to the variance of the Poisson distribution. 

We perform negative binomial regression analysis with the intensity of visit as the outcome variable and predisposing, enabling, and need factors as explanatory variables

(4) $$\lambda_{i}=\exp(X_{i}{}^{\prime }\beta ),$$

where **X** is the vector of covariates (age, sex, education, referral source, income, chronic status, discharge status, number of diagnoses, and primary diagnoses), and β is the vector of corresponding regression coefficients for clients i=1, 2,…,n. The negative binomial model is widely used in examining the factors affecting the intensity of clinic visits in the presence of overdispersed data [31,32,33]. The analysis was done in Stata 17.0 [34].

## 3. Results

### 3.1. Telehealth Visit Trends

**Figure 1:**
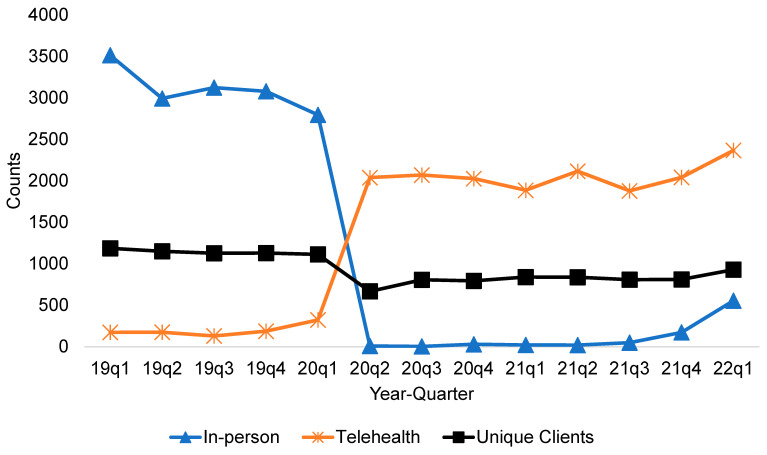
Trends in quarterly in-person and telehealth outpatient mental health visits encountered in Northeast Delta Human Services Authority (NEDHSA) behavioral health clinics before and during the COVID-19 pandemic. Note: 19q1 represents 2019 quarter 1 (2019 January to March).

Figure 1 shows the trends of quarterly in-person and telehealth mental health visits before and during the COVID-19 pandemic encountered in NEDHSA outpatient clinics. The number of telehealth visits was only slowly increasing before the first quarter of 2020 and comprised approximately 10% of total visits. That trend changed drastically during the pandemic period, with an abrupt decline in in-person visits and sharp uptake in telehealth visits following Governor Edwards’s issuance of a stay-home order on 22 March 2020 to contain the spread of the virus. Consequently, in the second quarter of 2020 (April to June), the number of telehealth services increased by 531% compared to the first quarter of 2020 (January to March). Conversely, the number of in-person visits decreased by 99.68% in the second quarter of 2020. The rise in telehealth visits, however, was not sufficient to offset the decrease in in-person visits, resulting in a net decrease in total visits during the pandemic. The number of unique patients remained largely stationary until 2020-q1, which decreased after the stay-home order.

### 3.2. Descriptive Statistics 

**Figure 2:**
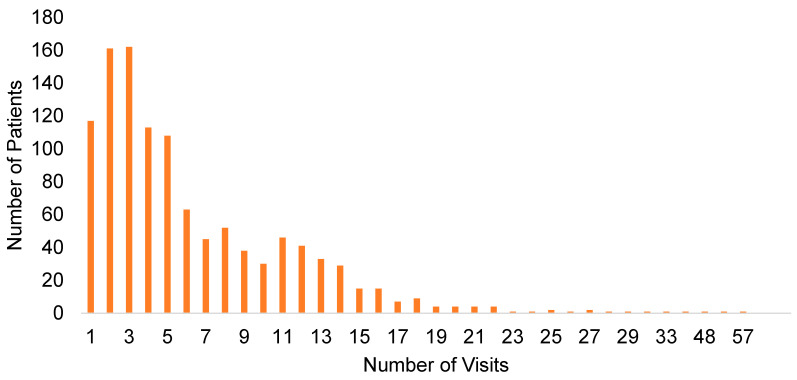
Frequency of telehealth visits for outpatient mental health services during the COVID-19 pandemic.

Our study included 7069 telehealth visits by 1115 unique patients from 1 April 2020 to 31 March 2021. Patients’ demographic and clinical characteristics are summarized in Table 2. The frequency of telehealth visits per patient during the study window ranged from 1 to 57 (Figure 2). More than 90% of patients utilized telehealth services less than 13 times. The mean and median number of visits were 6.34 and 5, respectively. The independent variables were grouped into predisposing, enabling, and needs factors. The majority of patients were 46–60 years old (33%), female (60%), and African American (55.2%). The average year of schooling was 11.5 years. Almost 50% of the patients were self-referred, whereas the rest were referred from the Federal Bureau of Prisons, clergy, city/parish/state courts, drug court, general hospital, inpatient psychiatric facilities, and nursing homes. The median monthly self-reported income was $790. Around 41% of the patients had at least one chronic health condition such as hypertension, cancer, and diabetes. The number of diagnosed mental illnesses ranged from 1 to 8, with an average of 1.722 diagnoses per patient. The most prevalent mental disorder was depressive disorder (37%), which was followed by schizophrenia spectrum and other psychotic disorders (32.2%). Discharged clients were those who were administratively discharged and completed treatment, referred elsewhere, did not follow up for appointments, lost contact, or died. Around 6% of the patients were discharged during the study window.

### 3.3. Regression Analysis

Table 3 shows the estimated Incidence Rate Ratio from the Negative Binomial regression model, reflecting the magnitude of associations between predisposing, enabling, and needs variables of healthcare utilization and telehealth visit counts. Being younger, female, and having received more education were associated with increased intensity of telehealth visits. Compared to the elder age group (>60 years), while holding other variables constant, the intensity of telehealth visits increased by 16.4%, 21.6%, and 22.3% in the 18–30, 31–45, and 46–60 years age groups, respectively. The intensity of visits was 11.3% higher in females compared to males. Similarly, years of schooling also had a positive and significant association with the intensity of telehealth visits. Some patients were discharged during the study period. Compared to the continuously enrolled patients, the intensity of visits for the discharged patients decreased by 45%. The presence of other chronic health conditions was positively associated with the intensity of telehealth visits, with about a 10% increase in visits compared to the patients without chronic conditions. The telehealth visits varied across the primary diagnosis type of patients. In particular, the intensity of telehealth visits among patients with schizophrenia spectrum and other psychotics decreased by 15% compared to patients with depressive disorders.

## 4. Discussion

The prevalence of mental health disorders among U.S. adults living in urban and rural areas is almost the same [9]. However, the utilization of mental health services among rural residents is lower than in urban populations [9]. While the under-utilization of in-person mental health services before the COVID-19 in the rural U.S. itself was a problem, fresh concerns have been raised about the effectiveness of pandemic-driven transition to telehealth services in providing adequate mental health services in the rural community because of the existing disparities in digital literacy and internet connectivity [35]. Our results suggest that although various push and pull factors, including the national and local policies on stay-at-home orders and relaxation of reimbursement policies, helped increase telehealth visits, they could not fully offset the decrease in in-person visits, resulting in a net decline in the total number of visits during the pandemic period.

To provide effective treatment and appropriate allocation of clinical resources, it is important to understand the factors that determine the utilization of mental health services. Decision making in the utilization of healthcare services and engagement in ongoing treatment is affected by various factors such as sociodemographic characteristics, health attitudes and beliefs, and physical and mental health status [19]. Poor engagement and a lower utilization rate of the mental health services may increase the treatment cost in the long run, aggravate additional burdens, and have no improvement on interpersonal and family functioning [36]. In addition to recognizing the mental health issue, accepting the intervention, enrolling in the treatment plan, and persistency in ongoing treatment and service utilization positively impact the outcome [37,38]. Conversely, treatment discontinuity without medical advice or formal discharge degrades the mental health status [39].

We find that several demographic and socioeconomic factors help determine the level of mental health service utilization. First, we find the intensity of telehealth service use among elderly patients to be lower. Although the intensity of mental health visits for the individuals in the older age group is higher [23,40], this pattern does not appear to hold with telehealth service utilization. The presence of disability and inexperience with digital technology use are some of the factors that keep elders from utilizing telehealth services [41]. We also find that the intensity of telehealth mental service use is significantly higher among females. This finding is consistent with the previous findings on health service use that suggest the prevalence of help-seeking behavior is higher in females compared to males [42,43]; however, our study extends these findings to the intensity of service use. The education level is a significant predictor of telehealth service utilization. Individuals with lower education levels may likely have poor health literacy, which is a major determinant of mental health treatment and service utilization [44]. A lesser level of education might translate into limited digital literacy, which is a key barrier to accessing telehealth services [45]. Consistent with the study of mental health service use [46] and intensity of mental health service use [23], our findings suggest that a higher education level is associated with the higher intensity of telehealth service use in mental health outpatient clinics.

With regard to the need factors in Anderson’s model, we find that several clinical factors are associated with the intensity of telehealth service use. Unsurprisingly, telehealth service use intensity is lower for discharged patients and higher for patients diagnosed with multiple mental health disorders. Moreover, individuals with chronic health conditions are more likely to suffer from mental illness compared to those not having chronic health conditions and may require more hospital visits [47,48]. For example, patients with diabetes have 60% and 123% higher odds of depressive and anxiety disorder, respectively [49]. Poor metabolic control, increased risk of complications, decreased quality of life, and medication adherence is strongly linked with mental health challenges [50]. Furthermore, patients with chronic health conditions may often receive advice from their primary care doctor to visit the mental health outpatient clinics, thereby increasing mental health visits. Our study is consistent with the study of Nour et al. [51], which suggests that decreased physical health functioning is associated with increased mental health service visits. Furthermore, we find that the intensity of telehealth visits is lower for individuals diagnosed with schizophrenic spectrum disorder and other psychotic disorders. While the past studies suggest that telehealth may be effective for various mental health diagnoses [52,53], the effectiveness of telemedicine in treating more severe mental health conditions such as schizophrenia and psychotic disorder remains in doubt. A recent article by Zhu et al. [18] examines the large claims data for mental health outpatient visits and reports that the service utilization pattern for schizophrenia conditions is lower in telehealth modalities compared to physical visits. Patients with schizophrenia may likely experience discomfort or challenges in engagement with the telehealth services and tend to ignore or miss the tele-visit.

There are several strengths of this study. First, we explore the factors associated with the intensity of outpatient mental health clinic visits in a rural setting during a period of a national health emergency. This is particularly important, as there is increasing use of telehealth services after the onset of the COVID-19 pandemic, while the studies on its effectiveness, especially among mental health patients in rural areas, remain limited. Second, unlike other studies that examine the intensity of clinic visits based on patients’ self-report, we did not use the self-reported survey data, which might be subject to recall bias or social desirability bias. Instead, we used the electronic health records of the patients encountered during an outpatient clinic visit, which likely offers a more objective picture of the use of health services. Our major limitation in the study is that we only considered the primary mental health diagnosis of the patient, even though there were many patients with multiple mental health conditions. Whereas we controlled for the number of diagnoses in the model, there remains the issue of identifiability of the effect of each single mental health diagnosis.

On the whole, our study speaks to the importance of removing barriers in telehealth service use among mental health patients living in rural communities. Because rural dwellers are affected by the existing digital divide, are more likely to be an older age group, are less likely to have education completed until high school, and are more likely to develop chronic health conditions [54], attention should be paid to the effectiveness of telehealth medicine, especially among the elderly, less educated, and those with serious mental health challenges such as schizophrenia. Future research comparing the intensity of mental health visits before and during the pandemic, and comparing telehealth service use in the rural and urban areas would be of interest.

## 5. Conclusions

Effective treatment of mental health conditions is largely dependent on the optimum utilization of healthcare services. The COVID-19 pandemic disrupted traditional in-person medical care, and health providers adapted to telehealth services. This study examined the trend of telehealth visits and explored the factors associated with the intensity of service use among rural dwellers. Using Anderson’s behavioral model of healthcare utilization as the theoretical framework, we find that several predisposing and need factors help determine the level of telehealth service use. The elderly, less educated, and mental health patients with schizophrenia are less likely to use telehealth services. Policies targeted to reduce the digital divide and tailored to specific sub-classes of mental health illnesses could be helpful in promoting telehealth services to rural residents.

## Figures and Tables

**Table 1:** Characteristics of parishes in Louisiana.

Variables	Parishes Included in the Study (N = 12) Mean (S.D.)	Rest of the Parishes in Louisiana (N = 52) Mean (S.D.)
Population size	29,070 (41,132)	82,995 (105,523)
Proportion of households with internet subscription	0.62 (0.12)	0.76 (0.08)
Median household income in the past 12 months	37,371 (6360)	48,549 (11,480)
Proportion of population without health insurance	0.09 (0.02)	0.09 (0.01)
Proportion of population aged 65 years and above	0.18 (0.03)	0.16 (0.02)
Proportion of Black population	0.39 (0.17)	0.30 (0.14)
Proportion of non-Hispanic White population	0.57 (0.17)	0.62 (0.13)
Proportion of ≥25 years population with a minimum of college education	0.17 (0.07)	0.18 (0.07)

Note: Only 62% of the study region’s population had access to the internet, which is lower than the other counties of LA. Source: American Community Survey 5-year estimates 2016–2020.

**Table 2:** Characteristics of the sample (N = 1115).

	Variable	Number (N)	Proportion (S.D.)
Dependent variable			
	Number of visits (mean, S.D.)		6.34 (5.635)
Independent variables			
Predisposing factors	Age (years)		
	18–30	255	0.229
	31–45	322	0.289
	46–60	368	0.330
	60 and above	170	0.152
	Gender (female)	623	0.559
	Education (years of school, mean, SD)		11.50 (5.36)
	Referral source (self)	550	0.493
	Race		
	African American	615	0.552
	White	482	0.432
	Others	18	0.016
Enabling factor	Monthly income ($) (mean, SD)		845 (887)
Needs factors	Discharge (yes)	69	0.062
	Chronic condition (yes)	461	0.413
	Number of diagnoses (mean, SD)		1.722 (0.961)
	Diagnosis type		
	Anxiety disorders	44	0.039
	Bipolar & related disorders	158	0.142
	Depressive disorders	413	0.370
	Other mental health challenges	94	0.084
	Schizophrenia spectrum & other psychotic disorders	359	0.322
	Trauma & stressor related disorders	47	0.042

Note: The number of visits is the dependent variable. Independent variables are grouped into predisposing, enabling, and needs factors.

**Table 3:** Incidence Rate Ratio from Negative Binomial regression analysis.

Variable	Incidence Rate Ratio	Standard Error
Age in years (Ref: >60)		
18–30	1.164 *	0.094
31–45	1.216 ***	0.091
46–60	1.223 ***	0.089
Gender (female, Ref: Male)	1.113 **	0.055
Number of school years	1.010 **	0.005
Referral source (self, Ref: external sources)	0.998	0.048
Race (Ref: White)		
African American	0.991	0.049
Others	0.741	0.142
Monthly income (in thousand USD)	1.029	0.027
Discharge (yes, Ref: No)	0.550 ***	0.058
Chronic condition (yes, Ref: No)	1.101 **	0.054
Number of diagnoses	1.067 ***	0.027
Primary diagnosis type (Ref: Depressive disorder)		
Anxiety disorders	0.959	0.117
Bipolar and related disorders	0.903	0.065
Other mental health challenges	0.928	0.084
Schizophrenia spectrum and other psychotic disorders	0.850 ***	0.051
Trauma and stressor-related disorders	1.016	0.119

Notes: *** *p* < 0.01, ** *p* < 0.05, * *p* < 0.1.
